# PtdIns4P-mediated electrostatic forces influence S-acylation of peripheral proteins at the Golgi complex

**DOI:** 10.1042/BSR20192911

**Published:** 2020-01-06

**Authors:** Sabrina Chumpen Ramirez, Micaela R. Astrada, Jose L. Daniotti

**Affiliations:** 1 CONICET. Universidad Nacional de Córdoba, Centro de Investigaciones en Química Biológica de Córdoba (CIQUIBIC), Córdoba, Argentina; 2 Universidad Nacional de Córdoba, Facultad de Ciencias Químicas, Departamento de Química Biológica Ranwel Caputto, Córdoba, Argentina

**Keywords:** Endomembrane trafficking, Golgi complex, Palmitoylation, Palmitoyltransferase, PtdIns4P, S-acylation

## Abstract

Protein S-acylation is a reversible post-translational modification involving the addition of fatty acids to cysteines and is catalyzed by transmembrane protein acyltransferases (PATs) mainly expressed at the Golgi complex. In case of soluble proteins, S-acylation confers stable membrane attachment. Myristoylation or farnesylation of many soluble proteins constitutes the initial transient membrane adsorption step prior to S-acylation. However, some S-acylated soluble proteins, such as the neuronal growth-associated protein Growth-associated protein-43 (GAP-43), lack the hydrophobic modifications required for this initial membrane interaction. The signals for GAP-43 S-acylation are confined to the first 13 amino acids, including the S-acylatable cysteines 3 and 4 embedded in a hydrophobic region, followed by a cluster of basic amino acids. We found that mutation of critical basic amino acids drastically reduced membrane interaction and hence S-acylation of GAP-43. Interestingly, acute depletion of phosphatidylinositol 4-phosphate (PtdIns4P) at the Golgi complex reduced GAP-43 membrane binding, highlighting a new, pivotal role for this anionic lipid and supporting the idea that basic amino acid residues are involved in the electrostatic interactions between GAP-43 and membranes of the Golgi complex where they are S-acylated.

## Introduction

Protein S-acylation, also known as palmitoylation, consists of the addition of a lipid molecule to one or more cysteine residues through a thioester bond. This modification, which is widespread in eukaryotes, allows the reversible association of peripheral proteins with membranes or, in the case of integral membrane proteins, modulates their behavior within the plane of the membrane due to their partition to different micro- and nanodomains via interaction with other proteins and lipids [1–3].

S-acylation is mediated by protein acyltransferases (PATs) belonging to the zinc finger DHHC (aspartate-histidine-histidine-cysteine) family [4,5], whereas protein deacylation can be catalyzed by cytosolic acyl-protein thioesterases (APTs) belonging to the α/β-hydrolase family of serine hydrolases [6–11]. PATs are transmembrane proteins mainly localized at the Golgi complex, but have also been found in the endoplasmic reticulum and plasma membrane [4,12,13].

For many soluble proteins, myristoylation or farnesylation on specific amino acid residues provides sufficient lipophilicity for transient membrane adsorption prior to S-acylation, which strongly increases the binding to the lipid bilayer [14]. Thus, for instance, H-Ras is first farnesylated in the cytosol, thereby allowing transient association with the cytosolic surface of the endoplasmic reticulum and S-acylation to the Golgi complex [1,15,16]. However, many other S-acylated proteins do not contain specific receptors or membrane-targeting motifs, are not modified by the attachment of hydrophobic groups, and therefore lack the level of membrane affinity required to facilitate the initial membrane contact. Consequently, the precise molecular mechanisms involved in the binding of these proteins to specific organelles, such as the Golgi complex, for their S-acylation by membrane-associated PATs remains uncertain and needs to be fully elucidated.

Biophysical and biochemical studies have demonstrated the active participation of domains rich in basic amino acids in the electrostatic interaction between acylated proteins and anionic lipids of artificial and biological membranes [15,17–21]. Furthermore, it is known that palmitoylated cysteine residues are often preceded and/or followed by basic amino acids [22] and has been speculated that these positively charged residues bind negatively charged acyl-coenzyme A (donor acylation substrate), thus increasing the efficiency of acylation. We hypothesize that these residues could also be involved in the transient attachment of proteins to biological membranes containing PAT enzymes.

Growth-associated protein-43 (GAP-43), which is not modified in the cytosol by isoprenyl- or myristoyl groups, is a dually palmitoylated protein found at positions 3 and 4 of cysteine (C) residues. GAP-43 belongs to the class of intrinsically disordered proteins (IDPs) showing no signs of hydrogen-bonded secondary structure [23,24]. This protein localizes to the plasma membrane both in neural and non-neural cells. However, newly synthesized GAP-43 is first found mainly associated with the trans-Golgi network (TGN), where it is S-acylated probably by DHHC 7 and/or 17, trafficking then to the plasma membrane by vesicular transport [25]. Pharmacological inhibition of newly synthesized GAP-43 acylation or double mutation of GAP-43 C3 and C4 completely disrupts the TGN and plasma membrane association. However, it was found in COS-1 and PC12 cells that most of GAP-43 was depalmitoylated, but nevertheless membrane-bound suggesting that the palmitate chains of GAP-43 do not serve as a permanent membrane anchor [26,27]. Interestingly, the minimal domain necessary for GAP-43 TGN targeting has been mapped to amino acids 1–13 (^N13^GAP-43), comprising the two S-acylatable cysteines close to a cluster of positively charged amino acids [25,28,29].

In the present study, we explored the molecular mechanisms involved in the interaction of GAP-43 with TGN membranes prior to S-acylation, in particular the relevance of basic amino acids presents at the N-terminus of GAP-43 in the initial electrostatic adsorption of GAP-43 to TGN membranes via anionic phospholipids. By means of time-lapse imaging experiments we showed how acute depletion of negatively charged phosphatidylinositol 4-phosphate (PtdIns4P) from the TGN can regulate membrane binding and S-acylation of GAP-43. In the case of post-synaptic density protein PSD-95, a major protein in the brain palmitoylated at C3 and C5, we also found evidence of the early stage of protein–membrane interaction by electrostatic forces that precedes the stable membrane attachment mediated by S-acylation.

Overall, our results demonstrate a general molecular mechanism operating in the early membrane binding of S-acylatable peripheral proteins at the Golgi complex, highlighting a new, pivotal role for PtdIns4P, a key regulator of a wide range of cellular functions.

## Materials and methods

### Plasmids and constructions

The expression vectors pECFP-N1 (where ECFP is Enhanced Cyan Fluorescent Protein), pEYFP-N1 (where EYFP is Enhanced Yellow Fluorescent Protein) and pmCherry (mCh)-N1 were obtained from Clontech (Mountain View, CA, U.S.A.). Expression plasmids for GAP-43, ^N13^GAP- 43, ^N13^GAP-43(C3,4S) have all been previously described [25,30]. The mutants of ^N13^GAP-43 at basic residues were generated by PCR using ^N13^GAP-43 cloned in the pEYFP-N1 vector as a template and the appropriate primers (Supplementary Table S1). PSD-95 and the mutant versions were generated by PCR using cDNA from rat brain as a template and the appropriate primers (Supplementary Table S1). The plasmids expressing the rapamycin-inducible system (Tgn38-FRB-CFP and Sac1-FKBP12-mRFP) and the reporter [GFP-PH (pleckstrin homology) FAPP1 (PtdIns4P adaptor protein-1)] were kindly provided by Dr. Tamas Balla (National Institute of Child Health and Human Development, National Institutes of Health, Bethesda, U.S.A.) and are described in [31].

### Cell culture and transfection

Chinese hamster ovary (CHO)-K1 cells and CAD (Cath-a-differentiated) cells (ATCC, Manassas, VA, U.S.A.) were grown at 37°C, 5% CO_2_ in Dulbecco’s Modified Eagle Medium (DMEM) supplemented with 10% fetal bovine serum (FBS), 100 mg/ml penicillin and 100 mg/ml streptomycin. Cells grown on Petridishes were used for both live-cell imaging and Western blot experiments. At 80% confluence, cells were transfected with the indicated plasmid using linear polyethylenimine (PEI, Polysciences, Warrington, PA, U.S.A.) as described previously [16]. After cell transfection, cells were processed for Western blot experiments or plated on to Lab-Tek chambered coverglass (Thermo Scientific Nunc, IL, U.S.A.), incubated for the indicated time, and then used in live-cell imaging.

### Acyl-Biotin exchange

The Acyl-Biotin Exchange (ABE) method was carried out as described in [32] except that the samples were homogenates from 6-mm dishes with transfected CHO-K1 cells. Briefly, non-acylated cysteines were blocked with N-ethylmaleimide and samples were then incubated with neutral hydroxylamine to cleave thioester linked palmitate and allow HPDP-biotin reagent to react with the now free thiol group. As a control, half of the sample was incubated without hydroxylamine. Biotinylated proteins were purified with streptavidin-agarose beads and analyzed by SDS/PAGE and Western blot using antibodies against the protein of interest.

### 2-Bromopalmitate treatment

CHO-K1 cells were transfected in the presence of 60 μg/ml cycloheximide (CHX). Two hours after transfection, 50 μM 2-Bromopalmitate (2BP) was added and cells were incubated for 1 h at 37°C, 5% CO_2_. The medium was removed and fresh DMEM containing 10% FBS and 50 μM 2BP was added. Cells were incubated for 3 h before imaging by confocal fluorescence microscopy.

### Subcellular fractionation

CHO-K1 cells grown on 3.5-cm dishes were transfected with the indicated plasmid as described above. The growth medium was removed 15 h post-transfection and cells were washed with phosphate-buffered saline (PBS; 140 mM NaCl, 8.4 mM Na_2_HPO_4_, 1.6 mM NaH_2_PO_4_, pH 7.5) and lysed in lysis buffer (150 mM NaCl, 1 mM EDTA, 1 mM PMSF, Protease Inhibitor Cocktail (PIC, Sigma–Aldrich, MO, U.S.A.), 50 mM Tris/HCl pH 7.5). Homogenates were centrifuged for 5 min at 800×***g*** to remove the debris. The supernatants were centrifuged during 20 min at 17000×***g*** to collect the cytosolic (C) and membrane (M) fractions. Membranes were resuspended in lysis buffer containing 1% Triton X-100.

### SDS/PAGE and Western blotting

For protein detection, samples of the ABE assay or 40 μg of proteins from subcellular fractionation samples were prepared with the addition of Laemmli buffer 4× (Bio-Rad, Hercules, CA, U.S.A.) and run in 12% SDS/PAGE gels. Proteins were transferred to a nitrocellulose membrane and Western blot analysis was performed using the indicated primary antibodies. Antibodies were: anti-GFP (Roche Applied Science, Penzberg, Germany) 1:1000, anti-red fluorescent protein (RFP) (Invitrogen, CA, U.S.A.) 1/1500. The blots were probed using secondary antibodies coupled to either IRdye 680 or IRdye 800 (LICOR Bioscience, Cambridge, U.K.) at 1:20000 dilution, and then scanned using an Odyssey Infrared Imager (LICOR Bioscience, Cambridge, U.K.). Quantification and statistical analysis were carried out using ImageJ and GraphPad Prism software, respectively.

### Fluorescence microscopy

Cells grown on Lab-Tek II coverglass chambers were transfected with the indicated fluorescent constructs. Confocal images of live cells were collected 5 h post-transfection at 37°C (temperature and CO_2_ controller; Tokai Hit, Japan), using an Olympus FluoView FV-1000 laser-scanning confocal microscope equipped with an argon/helium/neon laser and a 63× PLAPON 1.4 numerical aperture oil-immersion objective (Olympus, Tokyo, Japan). Images were taken using a 4.5 digital zoom and single confocal sections of 0.8 μm. For Fluorescence Recovery After Photobleaching (FRAP) experiments, 100 nM rapamycin or vehicle (DMSO) was added to the culture medium 15 min before the photobleaching performed on the Golgi region using a 6× digital zoom, 10 μs/pixel, direct scan, 35% transmission of 405-nm laser during 5 min and a 63× UPlanApo oil immersion/1.42 NA objective. Under these conditions, YFP fluorescence after bleaching was <10% of its initial value. Pre-bleaching and post-bleaching images were obtained every 10 s (800 pixel × 800 pixel resolution) using the same objective. The fluorescence intensities of the bleached, pre-bleached and post-bleached areas were measured with ImageJ software (NIH, Bethesda, MD, U.S.A.).

## Results

### Highly conserved signals present in the first 13 residues of GAP-43 direct its S-acylation and subcellular distribution

**Figure 1:**
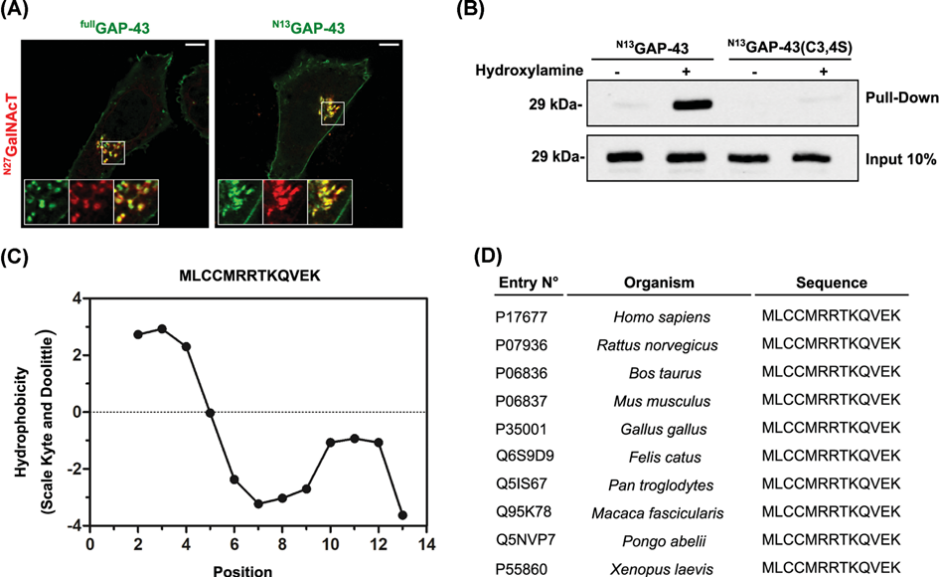
Characterization of the N-terminal motif of GAP-43 (**A**) CHO-K1 cells transiently transfected to co-express ^full^GAP-43-YFP or the ^N13^GAP-43-YFP motif and the TGN marker ^N27^GalNAcT-mCherry (^N27^GalNAcT-mCh) show the subcellular localization of the full-length and truncated version of GAP-43. Cells were imaged by live-cell confocal microscopy 5 h post-transfection and representative images are shown. The fluorescence signal corresponding to YFP fused proteins and that corresponding to the TGN marker are pseudocolored green and red, respectively. The individual and merged signals at the TGN region are shown in the boxed area at higher magnification. Scale bar, 5 μm. (**B**) ABE assay of ^N13^GAP-43-YFP and ^N13^GAP-43(C3,4S)-YFP expressed in CHO-K1 cells at steady state. Proteins from samples treated with Hydroxylamine (+) or the control Tris/HCl (−) were subjected to Western blotting with an antibody to GFP. (**C**) Hydrophobicity plot (Kyte–Doolittle scale) showing the polarity along the amino acid sequence of ^N13^GAP-43. (**D**) Schematic representation of the amino acid sequences showing the conservation of the N-terminal motif of GAP-43 between orthologs.

In contrast with many S-acylated substrates (i.e. H-Ras), the mechanisms mediating the interaction between GAP-43 and PAT enzyme-containing membranes have still not been elucidated. In a previous paper, we reported that ^N13^GAP-43 achieves the same subcellular distribution as the full-length protein [25]. Analysis of GAP-43 and ^N13^GAP-43 localization showed that both localized at the same compartment as the TGN marker ^N27^GalNAcT (β1-4 N- acetylgalactosaminyltransferase) and also at the plasma membrane (Figure 1A), suggesting that the minimal requirements for S-acylation are contained in the first 13 amino acids of this protein. Using a modified, specific version of the ABE assay for identifying S-acylated proteins, we confirmed that ^N13^GAP-43 is S-acylated in CHO-K1 cells, whereas as expected, the mutant ^N13^GAP-43(C3,4S) is not modified (Figure 1B). Taking these observations into account, we focused on exploring the mechanisms involved in the initial events of membrane adsorption of ^N13^GAP-43 before it becomes S-acylated. This motif contains two acylatable cysteines (C3 and C4) embedded in a hydrophobic region, followed by a cluster of basic residues (R6, R7, K9, and K13) that confer a positive net charge of +3, owing to the presence of a glutamic acid (E) residue at position 12. A hydrophobicity analysis of the first 13 amino acids by the Kyte–Doolittle method revealed a sharp distinction between the hydrophobic and polar regions (Figure 1C), suggesting differential, possibly synergistic roles for membrane binding and later S-acylation. To investigate the significance of this finding, we performed a sequence comparison between orthologs and found that the amino acids contained in ^N13^GAP-43 are 100% identical in mammals, birds, and amphibians (Figure 1D). The fact that the N-terminal motif is highly conserved among species strongly suggests an important role in the process of GAP-43 S-acylation.

### Positively charged amino acid residues regulate S-acylation of GAP-43

**Figure 2:**
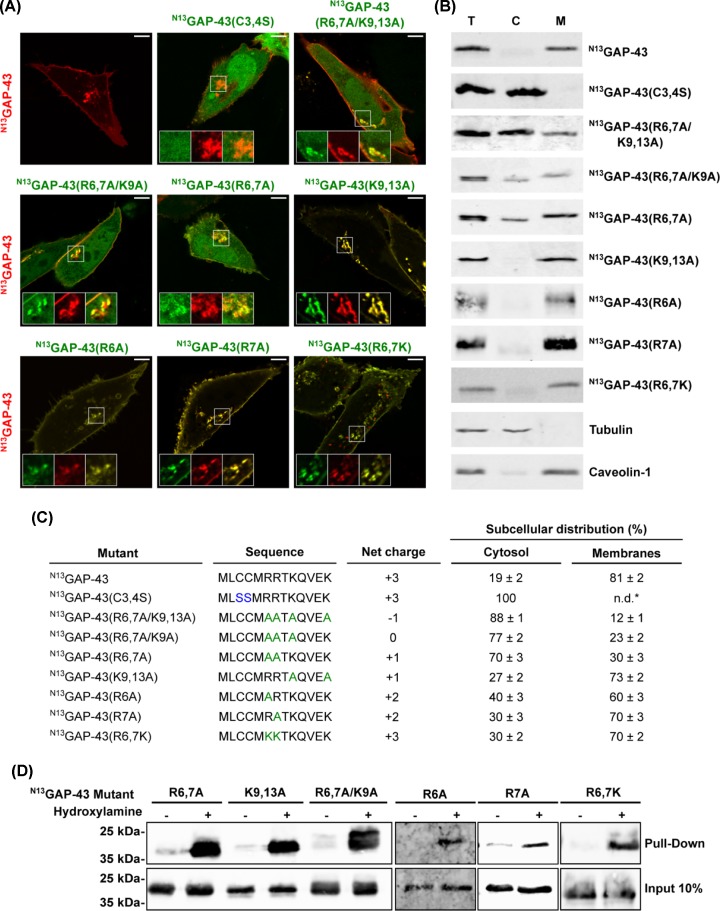
S-acylation, membrane association and subcellular localization of ^N13^GAP-43 mutants in basic residues (**A**) CHO-K1 cells transiently transfected to co-express the ^N13^GAP-43-mCh and the indicated mutant versions of ^N13^GAP-43 fused to YFP. Cells were imaged by live-cell confocal microscopy 5 h post-transfection. Representative images show the subcellular localization of the mutants compared with the non-mutated version of the N-terminal motif of GAP-43. The fluorescence signal corresponding to the YFP fused mutants is pseudocolored green and that corresponding to ^N13^GAP-43 is pseudocolored red. The TGN and plasma membrane regions are shown in the boxed area at higher magnification. Scale bar, 5 μm. (**B**) CHO-K1 cells transfected to express the indicated versions of ^N13^GAP-43-YFP were lysed and centrifuged to separate the cytosol (C) and membrane (M) fractions. Proteins from these fractions and the total homogenate (T) were subjected to Western blotting with an antibody to GFP. Tubulin and Caveolin-1 are shown as control markers of cytosol and membrane fractions, respectively. (**C**) Schematic representation of the amino acid sequences of wild-type ^N13^GAP-43, the non-acylatable mutant and the mutants in basic residues. The resulting net charge for each peptide is indicated. Quantification of results shown in (A) are also shown. The mean of the relative distribution in cytosol and membranes ± SEM is indicated (*n*=20). (**D**) ABE assay of the ^N13^GAP-43-YFP mutants in basic residues. Proteins from samples treated with Hydroxylamine (+) or the control (−) were subjected to Western blotting with an antibody to GFP.

The fact that a basic cluster is highly conserved at the N-terminal (N13) motif of GAP-43 led us to hypothesize that electrostatic interactions could mediate the initial absorption of this protein to the membranes, to be later modified by PATs. To investigate the role of basic residues, we generated several alanine (A) substitutions (Figure 2C) and compared their subcellular localization both with wild-type (^N13^GAP-43) and mutant [^N13^GAP-43(C3,4S)] proteins. CHO-K1 cells transiently transfected to express ^N13^GAP-43 or each of the mutants was analyzed by confocal fluorescence microscopy. As shown in Figure 1A, ^N13^GAP-43 localized in the perinuclear region (identified as TGN), plasma membrane and recycling endosomes, whereas the mutant ^N13^GAP- 43(C3,4S) remained completely cytosolic (Figure 2A). Imaging of mutants showed that mutation of all basic residues drastically reduced the membrane association of the ^N13^GAP-43(R6,7A/K9,13A) protein, which was found largely accumulated in the cytosol, with a minor fraction associated with the Golgi complex and completely absent from the plasma membrane (Figure 2A,C). This result demonstrates that all or most of the basic residues located in the N13 motif significantly contribute to the attachment of GAP-43 to membranes. The net negative charge (−1) of this mutant at the N13 motif could therefore interfere with the electrostatic interactions required for adsorption to the Golgi membranes. To determine the minimal number of positive charges required for ^N13^GAP-43 to be efficiently S-acylated and subsequently distributed in subcellular membranes, we characterized the neutral mutant ^N13^GAP-43(R6,7A/K9A) (see Figure 2C) and the double mutants ^N13^GAP-43(R6,7A) and ^N13^GAP-43(K9,13A), whose net charge is +1. Interestingly, the mutation of arginine residues—but not that of lysine residues—caused a noticeable accumulation of the mutants ^N13^GAP-43(R6,7A) and ^N13^GAP-43(R6,7A/K9A) in the cytosol (Figure 2A,C). It is important to remark that the cytosolic accumulation of these mutants was more evident at shorter than longer time periods of protein expression, possibly because of the accumulation of S-acylated proteins over long-time periods (i.e. microscopy versus Western blot analysis). The results suggest that the proximity of the positively charged residues to the S-acylatable cysteines (at least three positions) is a critical requirement for S-acylation. In order to further evaluate the role of individual arginines (R6 and R7), we constructed and characterized the single mutants ^N13^GAP-43(R6A) and ^N13^GAP-43(R7A), and the double mutant ^N13^GAP-43(R6,7K). Imaging of single mutants revealed that either of these residues (R6 and R7) are critical for S-acylation, since both mutants distributed to the same compartments as ^N13^GAP-43 (Figure 2A). The double mutant ^N13^GAP-43(R6,7K) also distributed to the same compartment as ^N13^GAP-43, confirming that the physical properties of amino acids at positions 6 and 7 are more critical for the efficient S-acylation of the N13 motif than the amino acid itself. In order to quantify the amount of cytosolic, deacylated protein fraction, we analyzed the extent of membrane association and the S-acylation status of these mutants by centrifuging extracts from mechanically lysed cells (Figure 2B). ^N13^GAP-43 was mainly associated with the particulate fraction. In contrast, ^N13^GAP-43(C3,4S) was completely associated with the soluble fraction. The single mutant ^N13^GAP-43(K9,13A) mostly partitioned in the pellet fraction, in accordance with the subcellular localization reported above, while a significant fraction of the triple mutant ^N13^GAP-43(R6,7A/K9A) was present in the soluble fraction after centrifugation. It should be taken into account that the soluble protein fraction could be underestimated due to the length of time required for expression in biochemical experiments. In agreement with the drastic change in the subcellular localization observed for ^N13^GAP-43(R6,7A/K9,13A), we found that most of the protein was present in the soluble fraction and only a minimal amount remaining attached to membranes, indicating that most of the mutant lack the lipid modification (Figure 2B). Using the ABE assay, we then corroborated that membrane association was effectively mediated by S-acylation of the mutants. Results show that all mutants were post-translationally modified at steady-state (Figure 2D) except for the ^N13^GAP-43(R6,7A/K9,13A) mutant, which could not be assessed because of the reduced expression of this mutant protein and/or low sensibility of the ABE assay. It should be mentioned that the ABE assay is an indirect and very useful method to demonstrate S-acylation of protein. However, it is not quantitative and complementary techniques are required to perform comparative and quantitative analysis.

**Figure 3:**
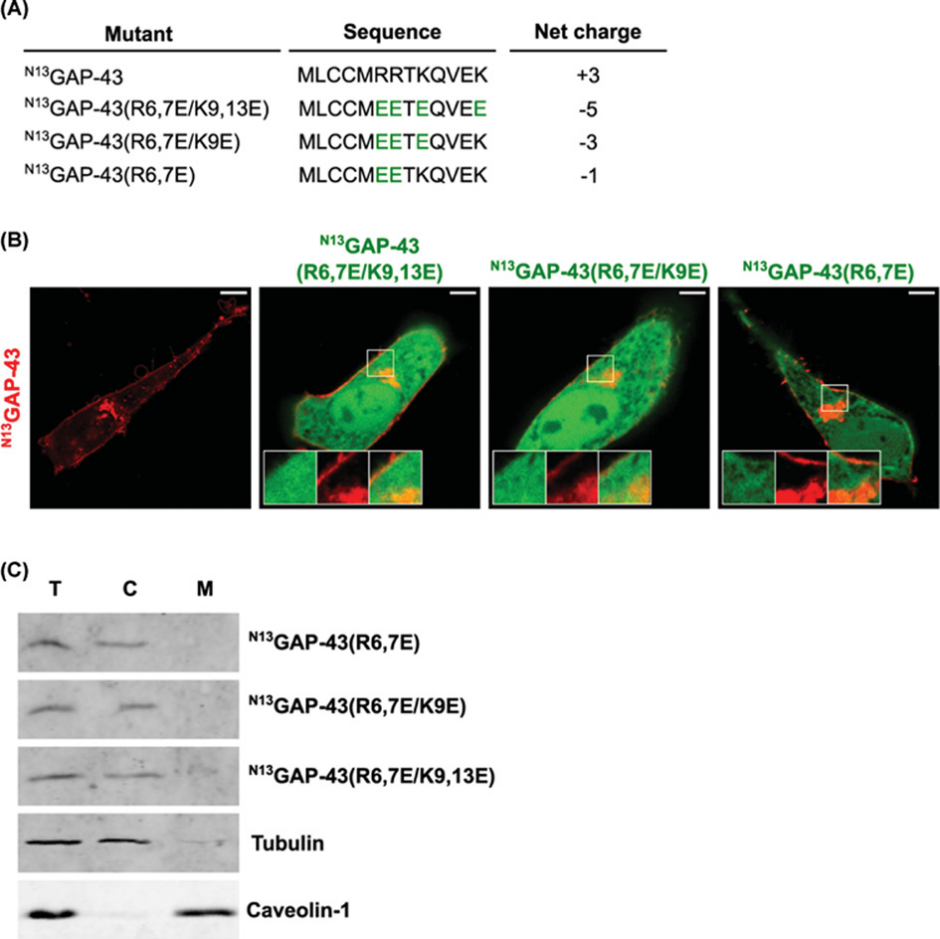
Membrane association and subcellular localization of ^N13^GAP-43(R6,7E), ^N13^GAP- 43(R6,7E/K9E) and ^N13^GAP-43(R6,7E/K9E) mutants (**A**) Schematic representation of the amino acid sequences of wild-type ^N13^GAP-43 and mutants. The resulting net charge for each peptide is indicated. (**B**) CHO-K1 cells transiently transfected to co-express the ^N13^GAP-43-mCh and the indicated mutant versions of ^N13^GAP-43 fused to YFP. Cells were imaged by live-cell confocal microscopy 5 h post-transfection. Representative images show the subcellular localization of the mutants compared with the non-mutated version of the N-terminal motif of GAP-43. The fluorescence signal corresponding to the YFP fused mutants is pseudocolored green and that corresponding to ^N13^GAP-43 is pseudocolored red. The TGN and plasma membrane regions are shown in the boxed area at higher magnification. Scale bar, 5 μm. (**C**) CHO-K1 cells transfected to express the indicated mutants were lysed and centrifuged to separate the cytosol (C) and membrane (M) fractions. Proteins from these fractions and the total homogenate (T) were subjected to Western blotting with an antibody to GFP. Tubulin and Caveolin-1 are shown as control markers of cytosol and membrane fractions, respectively.

To further support the hypothesis that electrostatic interactions could mediate the initial absorption of GAP-43 to the membranes, and trying to generate a more drastic effect due to the repulsion of the negative charges, we mutated the basic residues (R6,7 and K9,13) present in the N13 motif by acidic glutamic residues (^N13^GAP-43(R6,7E), ^N13^GAP-43(K9,13E) and ^N13^GAP-43(R6,7E/K9,13E) (Figure 3A). By biochemical and confocal microscopy analysis we found that all these constructs behaved as a soluble protein (Figure 3B,C), and the effect was more drastic to that obtained when arginine residues were replaced by neutral alanine residues, which strongly suggests that the interaction is mediated by electrostatic forces and not by a specific motif or structure present in the N-terminal domain.

**Figure 4:**
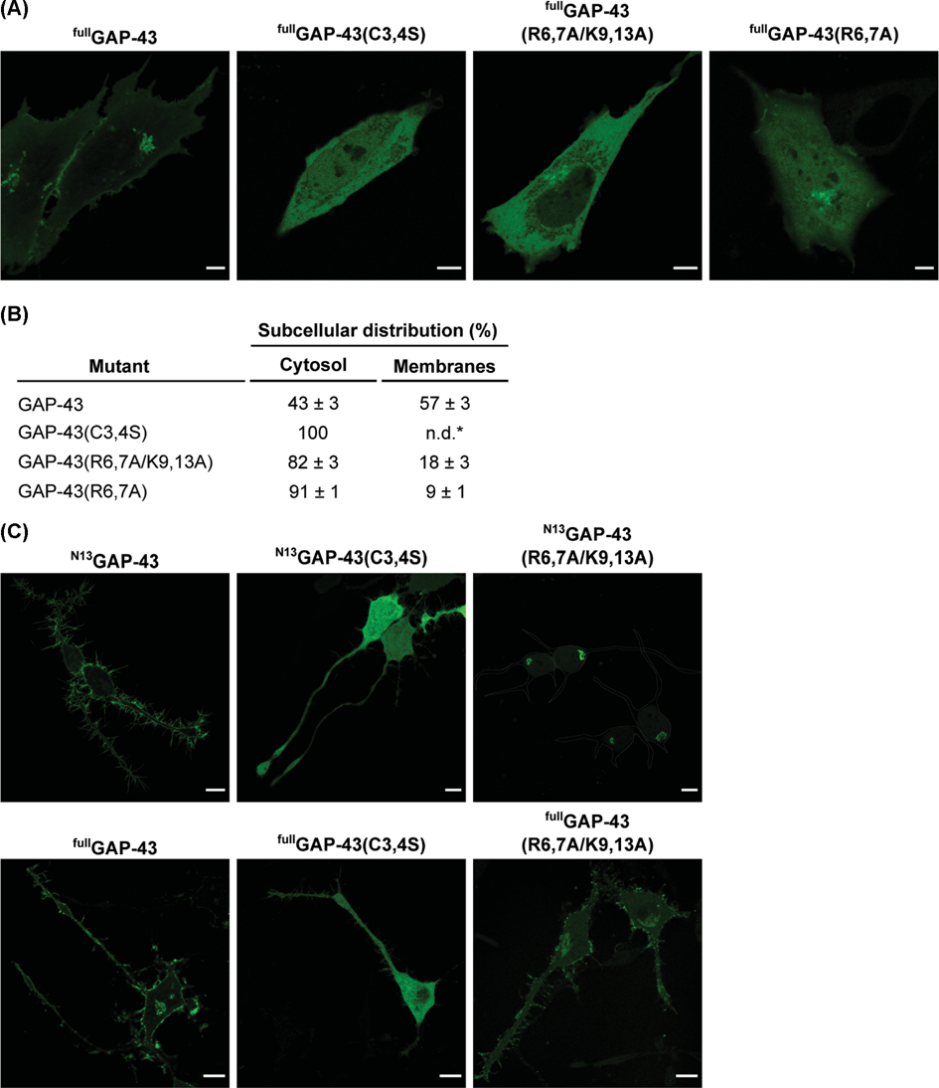
Subcellular localization of full-length GAP-43 mutants in basic amino acid residues both in epithelial and neuronal cells (**A**) CHO-K1 cells were transfected to express ^full^GAP-43 or the mutants ^full^GAP-43(C3,4S), ^full^GAP-43(R6,7A) or ^full^GAP-43(R6,7A/K9,13A) fused to YFP. Cells were imaged *in vivo* 5 h post-transfection by live-cell confocal microscopy. Scale bar, 5 μm. (**B**) Quantification of results shown in (A). The mean of the relative distribution in cytosol and membranes ± SEM is indicated (*n*=20). n.d., not detected. (**C**) CAD cells were transfected to express the ^N13^GAP-43 (upper panels) or ^full^GAP-43 (lower panels) and the indicated mutants fused to YFP. Cells were incubated 18 h post-transfection in the absence of FBS before live-cell imaging by confocal fluorescence microscopy. Top right, cell boundaries (white lines) are indicated. Scale bar, 10 μm.

We then sought to evaluate the role of basic residues in S-acylation in the context of the full-length GAP-43 (^full^GAP-43) protein. Our results indicate that the presence of arginines is the minimal requirement for efficient S-acylation of ^N13^GAP-43, but mutation of the four basic residues (R6, R7, K9, K13) drastically affected this lipid modification. Thus, we generated the ^full^GAP-43(R6, 7A) and ^full^GAP-43(R6,7A/K9,13A) mutants, fused to YFP, to further characterize their subcellular distribution in CHO-K1 cells. Confocal fluorescence imaging revealed that mutation of arginine residues caused an accumulation of the mutant in the cytosol (Figure 4A,B). As also reported for ^N13^GAP-43(R6,7A), a minor fraction of ^full^GAP-43(R6,7A) mutant was still associated with membranes, while the ^full^GAP-43(R6,7A/K9,13A) mutant was found mainly in the cytosol. Overall, these results indicate that signals present in the first 13 amino acids of GAP-43 are essential for its proper S-acylation.

In order to investigate whether the behavior of wild-type and mutant versions of GAP-43 in epithelial cells in regard to the role of basic amino acids in membrane binding is maintained in cells where GAP-43 is endogenously expressed, neuronal CAD cells were transfected to ectopically express ^N13^GAP-43, ^N13^GAP-43(C3,4S) or ^N13^GAP-43(R6,7A/K9,13A) and their subcellular localization analyzed. A similar analysis was also carried out using the full-length versions of both GAP-43 and mutants (Figure 4C). As observed in CHO-K1 cells, GAP-43 was membrane-associated, whereas the non-acylatable mutant was completely localized in the cytosol. The plasma membrane association of the mutant ^N13^GAP-43(R6,7A/K913A) was drastically reduced and a minor fraction remains associated with perinuclear membranes. The full-length mutant ^full^GAP-43(R6,7A/K9,13A) was able to associate with membrane, although an evident pool was observed in the cytosol (Figure 4C), suggesting inefficient S-acylation.

Taken together, these results demonstrate that the N-terminal motif is the main region involved in S-acylation of GAP-43 and that basic amino acid residues in the vicinity of the S- acylatable cysteines are key to the efficiency of the post-translational process.

### Basic amino acid residues around S-acylatable cysteines are not critical for adequate PAT–substrate recognition

**Figure 5:**
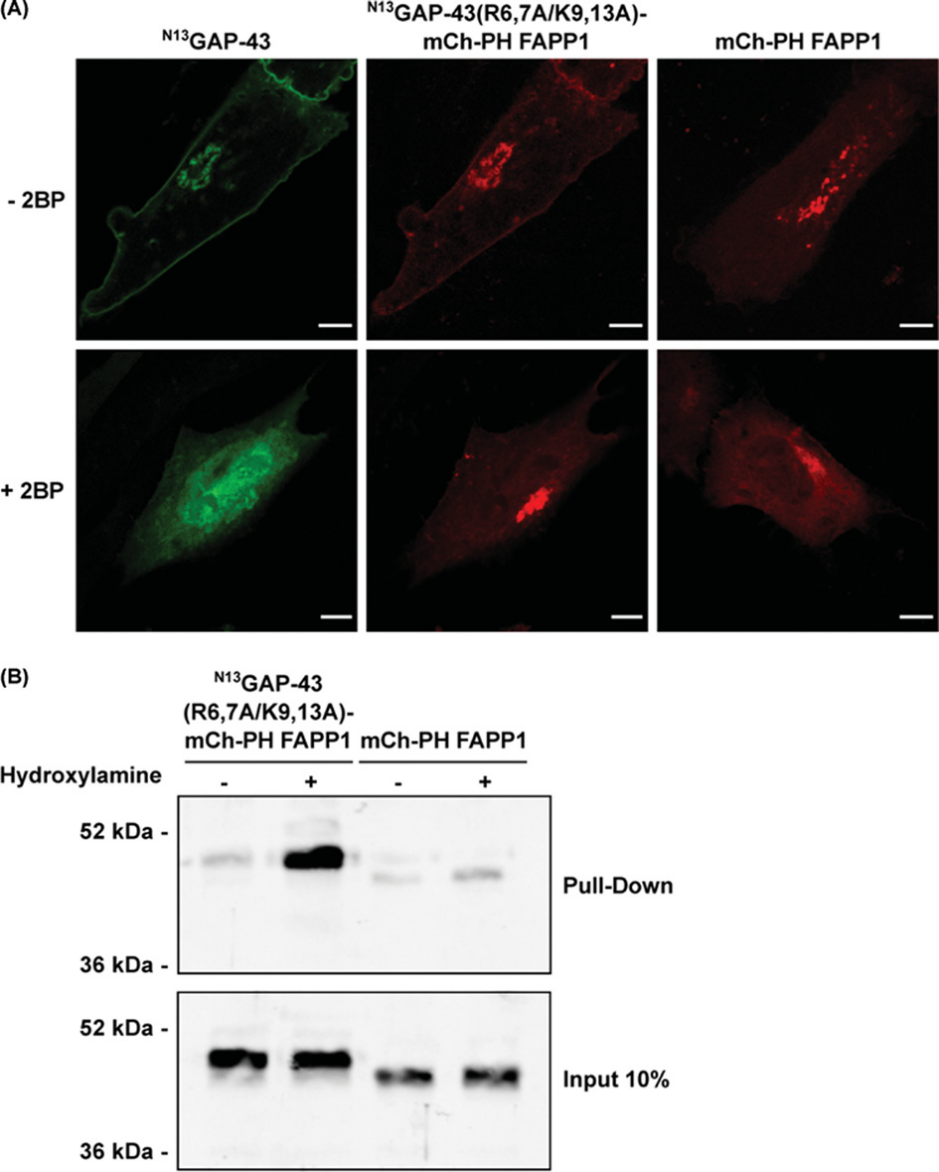
PtdIns4P-mediated binding of ^N13^GAP-43(R6,7A/K9,13A) protein to Golgi complex membranes Effect on S-acylation. (**A**) CHO-K1 cells were transfected to express the ^N13^GAP43-YFP and the chimera ^N13^GAP-43(R6,7A/K9,13A)-mCh-PH FAPP1 in the presence of 50 μM 2-bromopalmitate (+2BP) or vehicle (−2BP) and live-cell imaged by confocal microscopy 3 h post-transfection. As a control, cells expressing mCh-PH FAPP1 under the same conditions were also imaged. Representative images for each condition are shown. Scale bar, 10 μm. (**B**) ABE assay of the ^N13^GAP- 43(R6,7A/K9,13A)-mCh-PH FAPP1 expressed in CHO-K1 cells. The S-acylation status of mCh-PH FAPP1 was also assayed and included as a negative control. Proteins from samples treated with Hydroxylamine (+) or the control (−) were subjected to Western blotting with an antibody to RFP.

We next evaluated the possibility that the reduced S-acylation observed when basic residues are mutated could be a consequence of modifications in the PAT–substrate interaction. To test this, we designed a chimeric ^N13^GAP-43 construct able to bind the TGN membranes by a mechanism independent of electrostatic interactions, and we further characterized its subcellular localization and S-acylation status. The construct includes the ^N13^GAP-43(R6,7A/K9,13A) polypeptide fused to a fluorescent tag, mCherry (mCh), followed by the PH domain of the PtdIns4P adaptor protein-1 (FAPP1) (^N13^GAP-43(R6,7A/K9,13A)-mCh-PH FAPP1). The PH domain specifically interacts with PtdIns4P, the major phosphoinositide present in the TGN, forcing ^N13^GAP-43(R6,7A/K9,13A), found mainly in the cytosol, to interact with membranes where the PATs reside. CHO-K1 cells were co-transfected to express ^N13^GAP-43 and the PH chimera. Interestingly, ^N13^GAP-43(R6,7A/K9,13A)-mCh-PH FAPP1 was found completely anchored to membranes and localized at the same subcellular compartments as ^N13^GAP-43 (Figure 5A), suggesting that plasma membrane localization is directed by the ^N13^GAP-43(R6,7A/K9,13A) motif and probably depends on S-acylation. To explore this possibility in detail, CHO-K1 cell expressing both the PH chimera and ^N13^GAP-43 proteins were incubated in the presence of the PAT inhibitor 2BP (50 μM) and the subcellular localization was analyzed. 2BP should be used carefully to avoid erroneous interpretations since it is a non-specific inhibitor of S-acylation. Thus, 50 μM 2BP was the optimal and recommended concentration used in our experiments to avoid an effect on protein S-deacylation [33]. As shown in previous reports, blocking S-acylation caused the cytosolic localization of ^N13^GAP-43. However, as expected, the PH construct remained associated with the TGN membranes owing to the presence of the PH domain (Figure 5A). The construct expressing the PtdIns4P binding domain (mCh-PH FAPP1) was included as a control and was only detected at the TGN, both in the presence and absence of 2BP. Finally, the S-acylation status of ^N13^GAP-43(R6,7A/K9,13A)-mCh-PH FAPP1 was confirmed by the ABE assay, revealing that the chimera was modified at the cysteines in the N13 motif since the fusion protein containing only the fluorescent protein fused to the PH domain (mCh-PH FAPP1) was not S-acylated (Figure 5B). These results demonstrate that basic amino acid residues are not critical for the substrate–PAT interaction and acyl-group transference since the ^N13^GAP-43(R6,7A/K9,13A) is capable of being post-translationally modified; the results also confirm that trafficking to the plasma membrane is dependent on S-acylation.

### Acute manipulation of PtdIns4P affects the dynamics of GAP-43 S-acylation

We then attempted to identify the electronegative molecules present in membranes of the Golgi complex, which could be involved in the interaction with polybasic amino acids residues contained at the N-terminal region of GAP-43. The results described above reveal that PtdIns4P-interacting proteins are able to be S-acylated, suggesting the possible involvement (or non-interference) of this phosphoinositide in the post-translational process. We therefore sought to further investigate the role of PtdIns4P in the S-acylation of GAP-43 in mediating electrostatic interactions with the protein.

**Figure 6:**
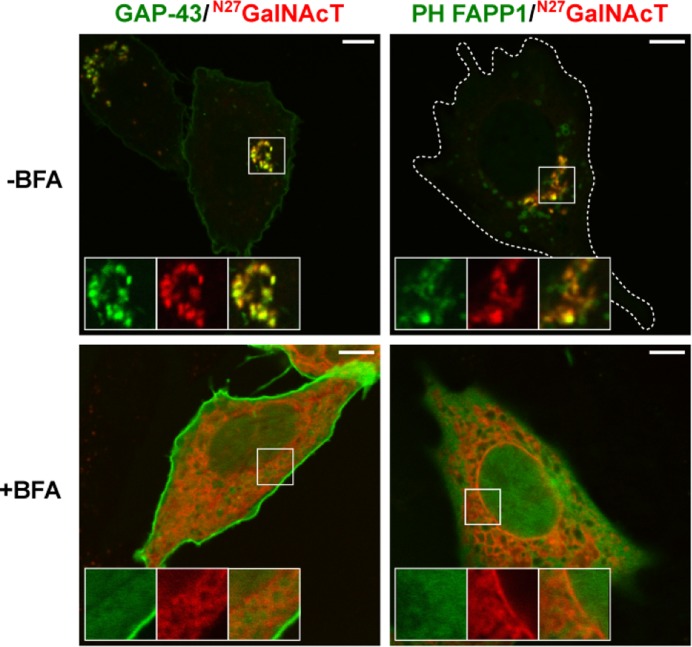
PtdIns4P depletion via treatment with BFA modifies the subcellular localization of GAP-43 protein CHO-K1 were co-transfected to transiently express GAP-43-YFP and ^N27^GalNAcT-mCh or GFP-PH FAPP1 and ^N27^GalNAcT-mCh, in the presence (+) or absence (−) of BFA. Three hours post-transfection, cells were live-cell imaged. Representative images show the effect of BFA on the membrane association of ^N13^GAP-43 and the PH domain of FAPP1. The fluorescent signals from YFP and GFP are pseudocolored green and the signal for mCherry (mCh) is pseudocolored red. The boxed area shows the TGN-associated proteins pre- and post-BFA treatment at higher magnification. Top right, cell boundaries (white lines) are indicated. Scale bars, 5 μm.

At the TGN, PtdIns4P is synthesized by PI(4)KIIα and PI(4)IIIβ [34]. Both enzymes are recruited by the GTPase Arf1, which is activated at the TGN by guanine nucleotide exchange factors, sensitive to Brefeldin A (BFA). Accordingly, we first evaluated the effect of BFA on the attachment of ^N13^GAP-43 to the TGN membranes. CHO-K1 cells were transfected during 3 h in the presence or absence of BFA to co-express ^N13^GAP-43 and the glycosyltransferase GalNAcT, a Golgi complex marker whose localization is independent of PtdIns4P. The PtdIns4P levels were monitored by expressing a fluorescent reporter fused to the PH domain of FAPP1. Under control conditions (-BFA), the PtdIns4P reporter (PH FAPP1) and ^full^GAP-43 co-localized with the Golgi complex marker, but the addition of BFA drastically affected their association with TGN membranes, increasing the expression in the cytosol (Figure 6). In addition, under BFA treatment, the Golgi complex membranes rapidly fused with the endoplasmic reticulum (ER) [35], as observed for the GalNAcT marker. These experiments strongly suggest that ^N13^GAP-43 S-acylation was reduced under conditions of PtdIns4P depletion via treatment with BFA.

**Figure 7:**
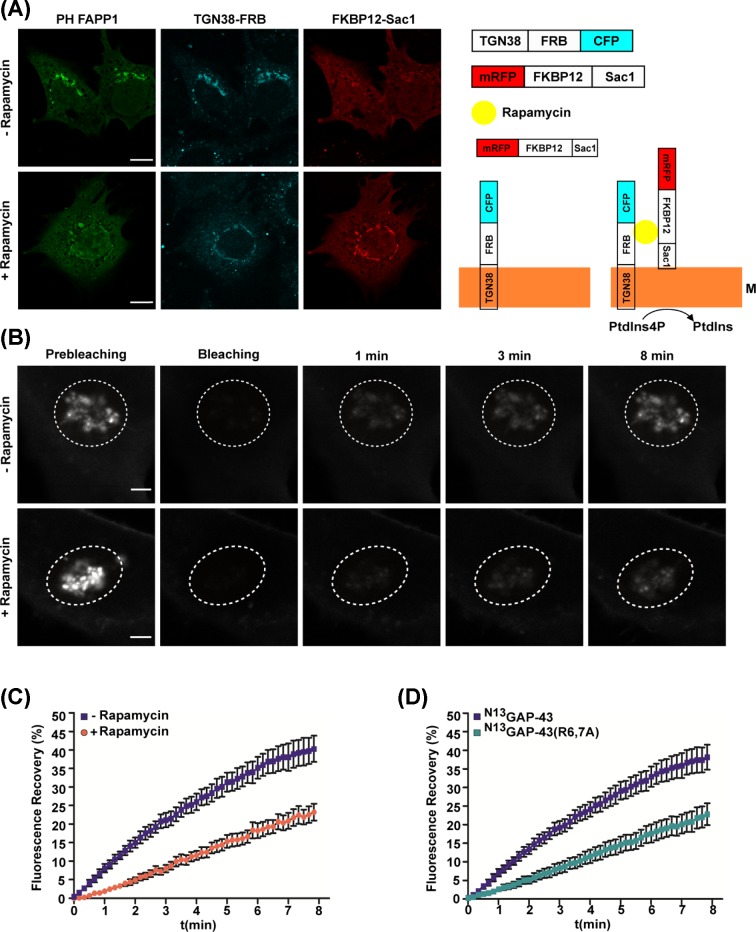
Effect of acute depletion of PtdIns4P on subcellular localization of ^N13^GAP-43 protein (**A**) CHO-K1 cells transiently expressing Tgn38-FRB-CFP, hSac1-FKBP12-mRFP and GFP-PH FAPP1 were incubated with 100 nM rapamycin (+) or vehicle (−) for 15 min and live-cell imaged by confocal fluorescence microscopy. Representative images show the rapamycin-inducible recruitment of hSac1 to the TGN membranes and the effect on membrane association of the reporter PH FAPP1. Scale bar, 5 μm. Panel on the right is a schematic representation of the rapamycin-inducible system. M represents the TGN membrane. (**B**) FRAP experiment. CHO-K1 cells were transfected to express ^N13^GAP-43-YFP, Tgn38-FRB-CFP and hSac1-FKBP12-mRFP. The experiment was performed 5 h post-transfection before or after incubation with 100 nM rapamycin. Representative images show the initial fluorescence at the TGN region, the photobleaching and the recovery of fluorescence at different times (1, 3, and 8 min). The dashed line indicates the photobleached area (ROI) corresponding to the TGN region. Images were taken every 10 s for 8 min after photobleaching. Scale bars, 3 μm. (**C**) Plot showing the mean of fluorescence recovery ± SEM (*n*=9 cells from two independent experiments) for the experiment described in (**B**). (**D**) Plot showing the mean of fluorescence recovery ± SEM (*n*=12 cells from three independent experiments) for ^N13^GAP-43-YFP and ^N13^GAP-43(R6,7A)-YFP after photobleaching. Abbreviation: ROI, region of interest.

To discard the possibility that the lack of S-acylation observed in the presence of BFA is due to secondary effects of this fungal metabolite, we used a direct system based on the rapamycin-inducible dimerization of the FKBP-12 protein and the FRB domain, which is able to recruit the phosphatase Sac1 to the TGN and thus specifically deplete PtdIns4P from this organelle [31]. Briefly, the system consists of two chimeric constructs: the FRB domain fused to CFP and to TGN38, a transmembrane protein localized in the TGN; and the FKBP-12 domain fused to RFP followed by the catalytic domain of Sac1, which localizes in the cytosol when expressed alone. PtdIns4P depletion in CHO-K1 cells was monitored using the PH FAPP1 fluorescent probe. As observed in Figure 7A, the addition of rapamycin caused the recruitment of Sac1 to the TGN concomitantly with dissociation of the reporter, which accumulated in the cytosol after 10–15 min of treatment.

We propose that electrostatic interactions promote S-acylation by anchoring GAP-43 to the TGN membranes, the efficiency of the process being reflected in the dynamics of the lipid modification. To quantify this, we measured the dynamics of ^N13^GAP-43 attachment to TGN membranes by FRAP experiments (Figure 7B). CHO-K1 cells were triple-transfected to express the inducible rapamycin system and ^N13^GAP-43. After 5 h of expression, a region of interest (ROI) including the entire Golgi complex was selected and bleached by laser irradiation for 5 s. The fluorescence recovery in the ROI was measured every 10 s during 8 min (Figure 7B and Supplementary Movie S1). We observed that the initial velocity of recovery and the total recovery of fluorescence after bleaching was drastically reduced under conditions of PtdIns4P depletion (+ Rapamycin) compared with the control condition (− Rapamycin) (Figure 7C).

We hypothesize that the reduction in negative charges at the TGN surface affected electrostatic interaction between the GAP-43 protein and membranes, reflected in reduced recovery. If this is the case, the mutation of basic amino acid residues should follow the same behavior. We therefore used FRAP experiments to analyze the membrane binding dynamics of the mutant ^N13^GAP-43(R6,7A) and compared them with those of wild-type ^N13^GAP-43. We found that ^N13^GAP-43(R6,7A) reached half the total recovery of the control condition. Moreover, the velocity of recovery was slower, as in the case of ^N13^GAP-43 dynamics when levels of PtdIns4P were reduced (Figure 7D).

**Figure 8:**
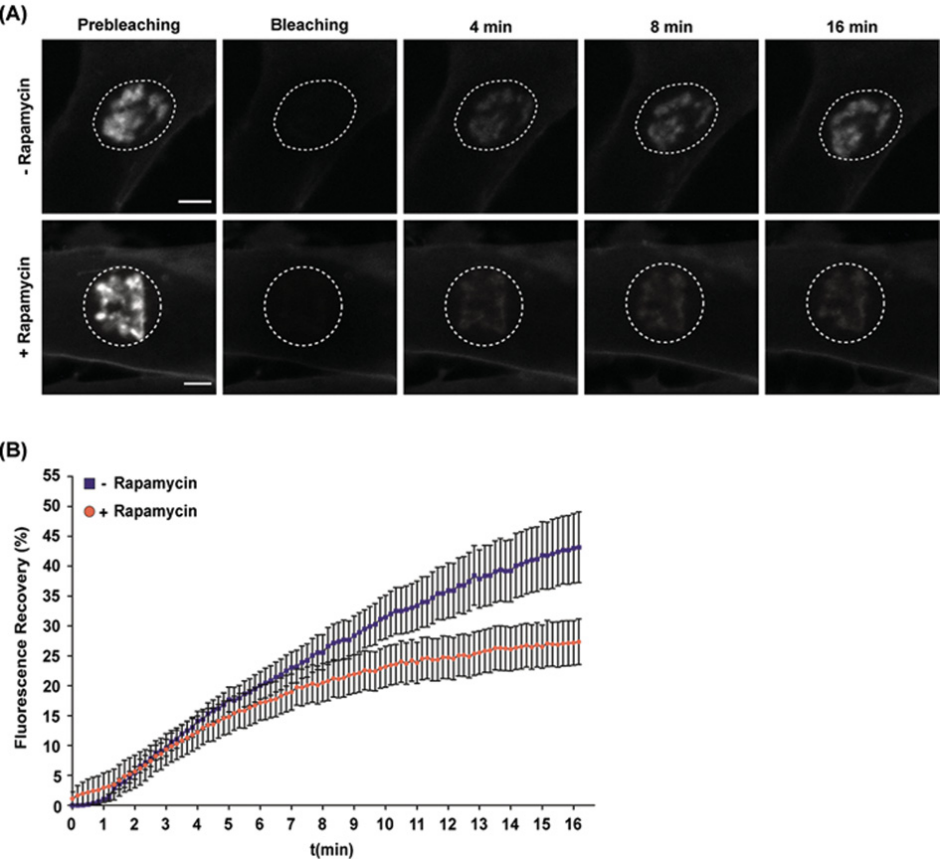
Effect of acute depletion of PtdIns4P on subcellular localization of full-length GAP- 43 protein (**A**) FRAP experiment. CHO-K1 cells were transfected to express ^full^GAP-43-YFP, Tgn38-FRB-CFP and hSac1-FKBP12-mRFP. The experiment was performed 5 h post-transfection before or after incubation with 100 nM rapamycin. Representative images show the initial fluorescence at the TGN region, the photobleaching and the recovery of fluorescence at different times (4, 8, and 16 min). The dashed line indicates the photobleached area (ROI) corresponding to the TGN region. Images were taken every 10 s for 16 min after photobleaching. Scale bars, 3 μm. (**B**) Plot showing the mean of fluorescence recovery ± SEM (*n*=17 cells from three independent experiments) for the experiment described in (A).

We then carried out similar FRAP experiments to evaluate the membrane-binding dynamics of ^full^GAP-43 protein. Although we observed a reduction in total recovery when PtdIns4P was depleted (+Rapamycin) (Figure 8A), the reduction in the initial velocity of recovery was not as drastic as for ^N13^GAP-43 (Figure 8B), suggesting the existence of additional factors affecting membrane interactions in the full-length protein.

Overall, these results strongly support our hypothesis that electrostatic interactions promote the initial event of adsorption of GAP-43 to TGN membranes and further demonstrate for the first time that a phosphoinositide is involved in S-acylation of proteins. Importantly, PtdIns4P dynamics could be responsible for a novel regulatory mechanism for the post-translational S- acylation of proteins.

### Basic amino acid residues surrounding S-acylatable cysteines probably define a general motif promoting the S-acylation of peripheral proteins

**Figure 9:**
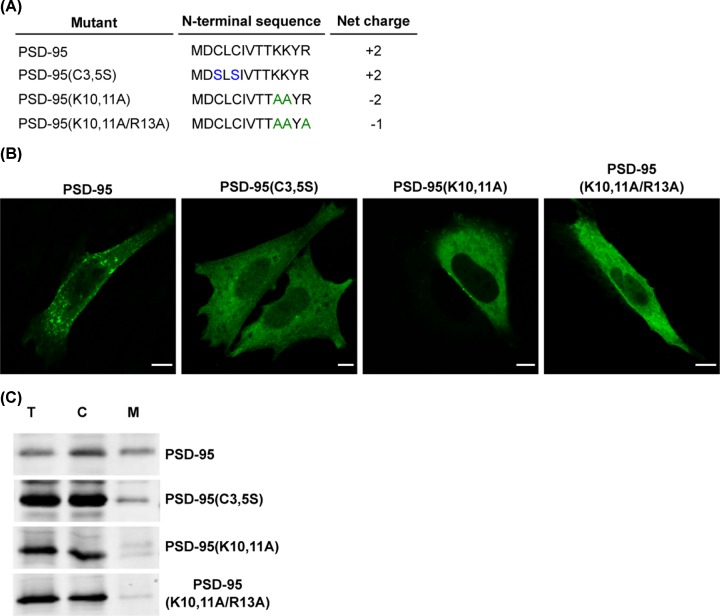
Membrane association and subcellular distribution of PSD-95 mutants in basic amino acid residues (**A**) Schematic representation of the amino acid sequences of the N-terminal motif of PSD-95, the non-acylatable mutant (C3,5S) and the mutants in basic residues. (**B**) CHO-K1 cells were transiently transfected to co-express the PSD-95 or the indicated mutants fused to YFP. Representative images show the subcellular localization of PSD-95 and the mutant versions. Scale bar, 5 μm. (**C**) CHO-K1 cells transfected to express the indicated versions of PSD-95 fused to YFP were lysed and centrifuged to separate the cytosol (C) and membrane (M) fractions. Proteins from these fractions and the total homogenate (T) were subjected to Western blotting with an antibody to GFP.

Since other S-acylated proteins share some features of the GAP-43 N-terminus (acylatable cysteines surrounded by hydrophobic residues followed by basic amino acids), it is possible that electrostatic interactions constitute an initial, general signal for the membrane anchoring of those proteins lacking farnesylation or myristoylation. To explore this hypothesis, we extended our analyses to the neuronal protein PSD-95, a peripheral protein localized at the Golgi complex, at endosomes and at a specialized post-synaptic membrane region called the post-synaptic density [36]. PSD-95 has two S-acylatable cysteines at the N-terminus (C3 and C5) and basic residues K10, K11, and R13 contained in a minimal sequence able to be diacylated (MDCLCIVTTKKYR). The role of these basic amino acid residues in the S-acylation of PSD-95 was assessed by generating and further characterizing the mutants PSD-95(K10,11A) and PSD-95(K10,11A/R13A) (Figure 9A). The subcellular localization of the mutants was analyzed in CHO-K1 cells and compared with the phenotype of the wild-type PSD-95 protein and the non-acylatable mutant PDS-95(C3,5S). We observed that PSD-95 was localized in the plasma membrane and also in intracellular structures, whereas PDS-95(C3,5S) localization was mainly in the cytosol (Figure 9B). Interestingly, mutation of K10 and K11 or K10, K11 and R13 resulted in clear accumulation in the cytosol and, in contrast with wild-type PSD-95, drastically reduced localization at the plasma membrane (Figure 9B). We next quantified the amount of soluble and membrane-associated fractions of these mutants by centrifuging extracts from mechanically lysed cells (Figure 9C). Wild-type PSD-95 protein equally distributed between soluble and particulate fractions. However, PSD-95(K10,11A) and PSD-95(K10,11A/R13A) mutant were mainly present in the soluble fraction, in accordance with the subcellular localization reported above.

These results, together with those obtained for the GAP-43 protein, strongly suggest that the presence of basic amino acid residues around S-acylatable cysteines might constitute a general requirement for promoting electrostatic interaction between peripheral proteins and cellular membranes, thus regulating the efficiency of protein S-acylation.

## Discussion

S-acylation is a post-translational lipid modification impacting on a wide variety of biological processes. Up to now, more than 2000 human proteins have been either shown or predicted to be S-acylated, representing approximately 12% of the proteome [37]. It is widely documented that S-acylation regulates the function of proteins by increasing their binding affinity to biological membranes, modifying their intracellular transport and subcellular distribution, affecting their stability and modulating their association with other proteins. However, the basic mechanisms involved in the early stages of membrane/protein contact, prior to S-acylation of peripheral proteins lacking membrane-targeting motifs or not modified by the attachment of hydrophobic groups, are still not fully understood.

In the present study, we demonstrate that a cluster of basic amino acids surrounding the two S-acylatable cysteines of GAP-43 are involved in the physical contact between the protein and Golgi complex membranes through an electrostatic mechanism. Results obtained via the systematic mutation of basic amino acids suggest that positive amino acids close to S-acylatable cysteine residues (R6 and R7) are more important for efficient membrane binding and S-acylation than those present at distal positions (K9 and K13). Previous studies also reported that a few basic amino acids in the cysteine-rich region of proteins are essential for transient plasma membrane targeting and that they are not critical for substrate–PAT interactions [26,27,38–41]. On the other hand, basic residues vicinal to the palmitoylated cysteines have also been reported to promote cysteine reactivity to nucleophilic attack and the interaction with the palmitate donor to increase the efficiency of cysteine palmitoylation [22,42]. In the context of these antecedents, we considered the possibility that the elimination of critical amino acids neighboring the cysteine residues of GAP-43 could perturb correct substrate-enzyme recognition in addition to cooperating in electrostatic binding with membranes. However, this possibility was discarded since we demonstrated that a mutated (net charge −1) and highly soluble version of ^N13^GAP-43 showed increased membrane targeting and S-acylation when forced to interact with Golgi complex membranes by fusion with the PH domain of the FAPP1 protein. This experiment led us to consider that PtdIns4P could participate in the membrane attachment of the short polybasic stretches of GAP-43 via electrostatic interaction rather than via a specific PH domain, this latter in fact being absent from the protein.

PtdIns4P is the most abundant of the monophosphorylated inositol phospholipids in mammalian cells. Though the bulk of this lipid is present in TGN membranes, the existence of extra-Golgi pools of PtdIns4P has been demonstrated [34]. At the Golgi complex, PtdIns4P participates in anterograde membrane trafficking at the exit of the organelle, and in sphingomyelin and glycosphingolipid metabolism [43]. To investigate the role of PtdIns4P in the membrane binding of S-acylatable proteins, we took advantage of an elegant approach to acutely deplete PtdIns4P via the rapamycin-induced recruitment of Sac1 phosphatase to TGN membranes [31].

On this basis we carried out FRAP experiments that demonstrated how the fast depletion of PtdIns4P at the TGN level drastically affected GAP-43 membrane binding, strongly suggesting the participation of this anionic lipid in the electrostatic binding between GAP-43 and Golgi membranes. Another interesting observation from these experiments is that the soluble fraction of GAP-43 does not appear to interact permanently with other cell endomembranes, which of course have sufficient electronegativity to support the binding but possibly lack the specific PATs to catalyze S-acylation and guarantee a more stable membrane interaction. Finally, experiments shown in Figure 2 indicate that a very small fraction of ^N13^GAP-43(R6,7A/K9,13A) protein remains associated with membranes in the perinuclear region, suggesting it had been S-acylated. However, the fact that the protein was not able to reach the plasma membrane is a possible indication that basic amino acids are also involved in the process of exiting the Golgi complex, perhaps by interaction with PtdIns4P. That the chimera ^N13^GAP-43(R6,7A/K9,13A)-PH FAPP1 was able to be acylated and localized at the plasma membrane would support this possibility, a point requiring further exploration.

As already mentioned, the first 13 amino acids of GAP-43 form an amphipathic motif containing the cysteine residues susceptible to S-acylation, followed by a region consisting of basic amino acids. Interestingly, this sequence was found to be 100% conserved in numerous species of mammals, birds and amphibians, strongly suggesting a fundamental role in the process of S- acylation of the protein. In addition to the basic net charge of the peptide, the hydrophobicity analysis weighted by the Kyte–Doolittle method shows that the polypeptide has a highly hydrophobic domain which together with the basic amino acids probably plays a significant, synergistic role in the interaction with membranes for subsequent S-acylation. Nevertheless, results shown in Figure 5 clearly indicate that basic amino acid residues surrounding S-acylatable cysteines are not required for the enzymatic process itself. These finding and assumptions are strongly supported by a recently developed predictor for S-Palmitoylation sites in proteins (SPalmitoylC-PseAAC) [44]. The software developers used Chou’s Pseudo Amino Acid Composition (PseAAC) and relative/absolute position-based features. The composition of amino acids surrounding the S-palmitoyl cysteine clearly showed a high predominance of positive amino acids (K, R) situated toward the carboxyl extreme, while hydrophobic amino acids (L, F) were present toward the amino extreme, but close to acylatable cysteine. Similar conclusions regarding preferred amino acids in the vicinity of palmitoylated cisteines were reported in a recently published work, which furthermore concluded that no conserved amino acid pattern could be associated with S-acylatable cysteines [45].

**Figure 10:**
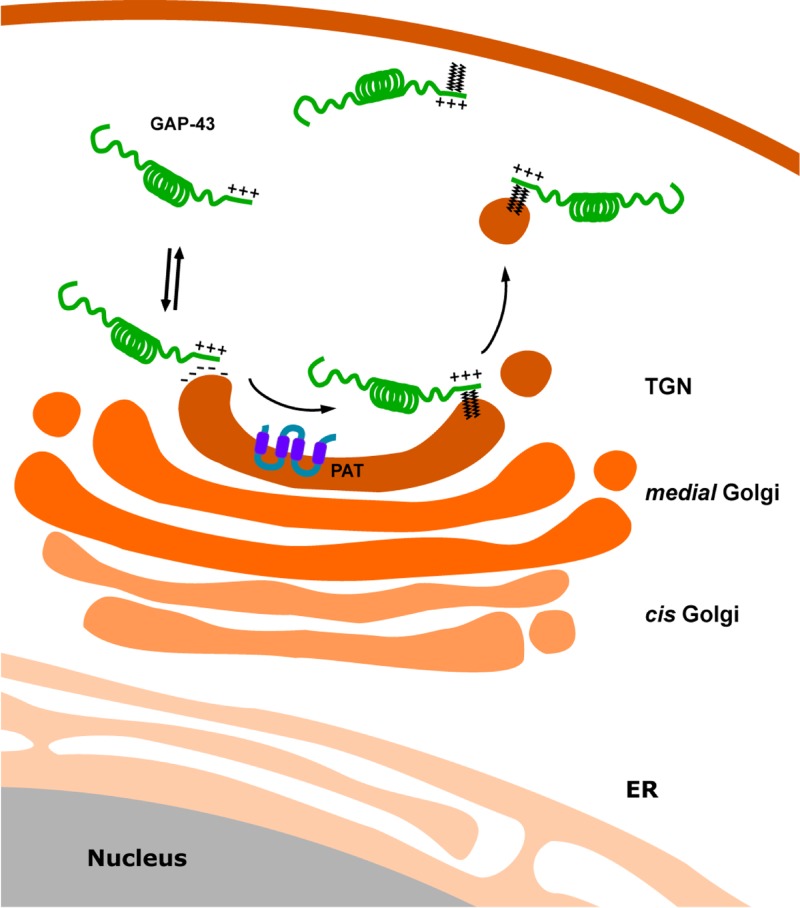
Schematic representation of the proposed model for membrane interaction and S-acylation of GAP-43 GAP-43 is synthesized in the cytosol. Electrostatic forces between basic residues present at the N- terminal motif of GAP-43 and negatively charged lipids (PtdIns4P) of the TGN membranes mediate the initial membrane interaction allowing the posterior interaction with the PAT. After S-acylation by the PAT at the Golgi complex, GAP-43 is included into the vesicular carriers directed to the plasma membrane.

Previous studies demonstrated the relevance of negatively charged phosphatidylinositol 4,5-bisphosphate [PtdIns(4,5)P2] and phosphatidylinositol 3,4,5-trisphosphate [PtdIns(3,4,5)P3] lipids to the regulated plasma membrane targeting of proteins containing clusters of polybasic amino acids [18,19,39]. Moreover, it was more recently reported that a few basic amino acids in the cysteine-rich region of SNAP25 and SNAP23 are essential for transient plasma membrane targeting, which precedes the stable membrane attachment mediated by S-acylation [41]. The data presented here provide a mechanistic model (Figure 10) to explain how peripheral S-acylatable proteins such as GAP-43 are targeted to the Golgi complex for S-acylation and highlight a new role of another polyphosphoinositide, PtdIns4P, whose finely tuned concentration might regulate S-acylation for a subset of peripheral proteins.

## Supplementary Material

Supplementary Table S1

Supplementary Movie S1

## Abbreviations

ABE: acyl-biotin exchange
BFA: Brefeldin A
CAD: Cath-a-differentiated
CHO: Chinese hamster ovary
DMEM: Dulbecco’s Modified Eagle Medium
FBS: fetal bovine serum
FRAP: Fluorescence Recovery After Photobleaching
GAP-43: growth-associated protein-43
mCh: mCherry
PAT: protein acyltransferase
PH: pleckstrin homology
PtdIns4P: phosphatidylinositol 4-phosphate
PSD-95: postsynaptic density protein-95
RFP: red fluorescent protein
ROI: region of interest
TGN: trans-Golgi network
