# Supplementary material for: PtdIns4P-mediated electrostatic forces influence S-acylation of peripheral proteins at the Golgi complex
Source: Biosci Rep. 2020 Jan 6;40(1):BSR20192911. doi: 10.1042/BSR20192911 (PMC6944663; doi:10.1042/BSR20192911)
Supplement: Supplementary Table S1 [file BSR-2019-2911_supp.pdf]

Supplemental Table 1: Oligonucleotides used in this study

**Oligonucleotide**	**Sequence**
5´ ^{N13}GAP-43(R6,7A/K9,13A)-YFP	AGCTCAAGCTTCGCCACCATGCTGTGCTGTATGGCAGCAACCGCACAGGTTGAAGCGGATCCACCGGTCGCCACCATGGTGAGCAAG
5´ ^{N13}GAP-43(R6,7A/K9A)-YFP	AGCTCAAGCTTCGCCACCATGCTGTGCTGTATGGCAGCAACCGCACAGGTTGAAAAAGATCCACCGGTCGCCACCATGGTGAGCAAG
5´ ^{N13}GAP-43(R6,7K)-YFP	AGCTCAAGCTTCGCCACCATGCTGTGCTGTATGAAAAAAACCAAACAGGTTGAAAAAGATCC
5´ ^{N13}GAP-43(R6,7A)-YFP	AGCTCAAGCTTCGCCACCATGCTGTGCTGTATGGCAGCAACCAAACAGGTTGAAAAAGATCCACCGGTCGCCACCATGGTGAGCAAG
5´ ^{N13}GAP-43(K9,13A)-YFP	AGCTCAAGCTTCGCCACCATGCTGTGCTGTATGAGAAGAACCGCACAGGTTGAAGCAGATCCACCGGTCGCCACCATGGTGAGCAAG
5´ ^{N13}GAP-43(R6A)-YFP	AGCTCAAGCTTCGCCACCATGCTGTGCTGTATGGCAAGAACCAAACAGGTTGAAAAGGATCCACCGGTC
5´ ^{N13}GAP-43(R7A)-YFP	AGCTCAAGCTTCGCCACCATGCTGTGCTGTATGAGAGCAACCAAACAGGTTGAAAAGGATCCACCGGTC
5´ ^{full}GAP-43(R6,7A)-YFP	CCGGAATTCCCACCATGCTGTGCTGTATGGCAGCAACCAAACAGGTTGAAAAGAATG
5´ ^{full}GAP-43(R6,7A/K9,13A)-YFP	CCGGAATTCCCACCATGCTGTGCTGTATGGCAGCAACCGCACAGGTTGAAGCGAATGATGAGGACCAAAAGATTG
5´ ^{N13}GAP-43(R6,7E/K9,13E)-YFP	TCGAGCTCAAGCTTCGCCACCATGCTGTGCTGTATGGAAGAAACCGAACAGGTTGAAGAGGATCCACCGGTCGCCACCATGGTGAGCAAG
5´ ^{N13}GAP-43(R6,7E/K9E)-YFP	AGCTCAAGCTTCGCCACCATGCTGTGCTGTATGGAAGAAACCGAACAGGTTGAAAAGGATCCACCGGTCGCCACCATGGTGAGCAAG
5´ ^{N13}GAP-43(R6,7E)-YFP	AGCTCAAGCTTCGCCACCATGCTGTGCTGTATGGAAGAAACCAAACAGGTTGAAAAGGATCC
3´ YFP N1 NotI-XbaI	TGATCTAGAGTCGCGGCCGCTTTACTTGTACAG
5´PSD-95	AAAGAATTCCCACCATGGACTGTCTCTGTATAGTG
5´PSD-95(C3,5S)	TTCGAATTCCCACCATGGACAGTCTCAGTATAGTGACAACCAAGAAATACCGC
5´PSD-95 (K10,11A)	AAAGAATTCCCACCATGGACTGTCTCTGTATAGTGACAACCGCCGCATACCGCTACCAAGATGAAGACACG
5´PSD-95(K10,11A/R13A)	AAAGAATTCCCACCATGGACTGTCTCTGTATAGTGACAACCGCCGCATACGCCTACCAAGATGAAGACACGCCCCC
3´PSD-95	TTTGTCGACTGGAGTCTCTCTCGGGCTGG
