# Supplementary material for: PtdIns4P-mediated electrostatic forces influence S-acylation of peripheral proteins at the Golgi complex
Source: Biosci Rep. 2020 Jan 6;40(1):BSR20192911. doi: 10.1042/BSR20192911 (PMC6944663; doi:10.1042/BSR20192911)
Supplement: Supplementary Movie S1 [file BSR-2019-2911_supp1.zip › BSR-2019-2911_suppV2.pdf]

Movie 1. Binding of ^{N13}GAP-43 to Golgi complex membranes.

Time-lapse video of CHO-K1 cells co-expressing ^{N13}GAP-43-YFP (pseudocolours reflect the intensity of the YFP signal) and the rapamycin-inducible system CFP-Tgn38-FRB and mRFP-FKBP12-hSac1 (not shown) imaged by live-cell fluorescence microscopy after photobleaching (as described in Figure 7). Movie is accelerated to 4 frames per second (4 fps). Note the difference in the recovery of ^{N13}GAP-43 on the Golgi complex in the absence (left panel, -Rapamycin) and presence (right panel, +Rapamycin) of rapamycin. Frames were acquired every 10 s for 8 min using a 63X/1.42 NA PlanApo objective oil immersion (Olympus, Tokyo, Japan). The movie is related to results shown in Figure 7B.
